# Novel uniplanar pedicle screw systems applied to thoracolumbar fractures: a biomechanical study

**DOI:** 10.3389/fbioe.2023.1172934

**Published:** 2023-05-30

**Authors:** Yuheng Jiang, Xiang Cui, Wei Ji, Jia Li, Yanli Shi, Jingxin Zhao, Junsong Wang, Peifu Tang, Wei Zhang

**Affiliations:** ^1^ Department of Orthopedics, Chinese PLA General Hospital, Beijing, China; ^2^ National Clinical Research Center for Orthopaedics, Sports Medicine and Rehabilitation, Beijing, China; ^3^ Department of Orthopedics, General Hospital of Southern Theater Command of PLA, Guangzhou, China; ^4^ Anesthesia and Operation Center, The First Medical Center of Chinese PLA General Hospital, Beijing, China

**Keywords:** biomechanical study, uniplanar pedicle screw, intermediate screw, thoracolumbar fractures, screw tulip design

## Abstract

**Objective:** In this study, the advantages of the internal fixation configuration composed of uniplanar pedicle screws in the treatment of thoracolumbar fractures were verified by biomechanical experimental methods, which provided the basis for subsequent clinical experiments and clinical applications.

**Methods:** A total of 24 fresh cadaveric spine specimens (T12-L2) were utilized to conduct biomechanical experiments. Two different internal fixation configurations, namely, the 6-screw configuration and the 4-screw/2-NIS (new intermediate screws) configuration, were tested using fixed-axis pedicle screws (FAPS), uniplanar pedicle screws (UPPS), and polyaxial pedicle screws (PAPS) respectively. The spine specimens were uniformly loaded with 8NM pure force couples in the directions of anteflexion, extension, left bending, right bending, left rotation, and right rotation, and the range of motion (ROM) of the T12-L1 and L1-L2 segments of the spine was measured and recorded to access biomechanical stability.

**Results:** No structural damage such as ligament rupture or fracture occurred during all experimental tests. In the 6-screw configuration, the ROM of the specimens in the UPPS group was significantly better than that of the PAPS group but weaker than those of the FAPS group (*p* < 0.01). In the 4-screw/2-NIS configuration, the results were identical to the biomechanical test results for the 6-screw configuration (*p* < 0.01).

**Conclusion:** Biomechanical test results show that the internal fixation configuration with UPPS can maintain the stability of the spine well, and the results are better than that of PAPS. UPPS has both the biomechanical advantages of FAPS and the superiority of easy operation of PAPS. We believe it is an optional internal fixation device for minimally invasive treatment of thoracolumbar fractures.

## 1 Introduction

Thoracolumbar fractures are a common consequence of external forces that result in continuous destruction of thoracic and lumbar vertebrae. With the increasing global aging population, the incidence of such fractures is on the rise. Pedicle screw and rod configuration is the most used surgical intervention. The surgery includes key steps such as fracture reduction and spinal stabilization. Fixed-axis pedicle screws (FAPS) have been the standard equipment for open internal fixation of the spine. However, during the operation of traditional open surgery, the soft tissue retraction may cause muscle crushing injury, destroy the muscle attachment point, *etc.*, which may cause postoperative pain and fatigue of the lower back muscles, extended recovery time, and in extreme cases, spinal function impairment.

In recent years, the use of posterior spine minimally invasive screw placement has become increasingly popular due to its effectiveness in addressing the limitations of traditional open surgery. By limiting the surgical approach’s breadth, minimally invasive surgery reduces soft tissue injury and the probability of postoperative low back pain and muscle weakening. FAPS must, however, be placed in the same plane and at the same depth during operation to allow for the connecting rod’s smooth insertion. The limited exposure of the minimally invasive surgical field and variations in surgeon expertise make inserting the connecting rod for FAPS challenging, resulting in increased operation time and variability in the operation’s outcome.

Surgeons are continuously seeking new pedicle screw fixation systems that are more convenient for minimally invasive surgery while maintaining biomechanical advantages. Among the available solutions, the polyaxial pedicle screw (PAPS) has been commonly employed. However, its overall biomechanics are weaker than those of fixed-axis pedicle screws (FAPS), resulting in the loss of vertebral body anterior height during the healing process (Yao et al., 2021). We must therefore create new pedicle internal fixation products that are stronger biomechanically and have superior therapeutic outcomes.

**FIGURE 1:**
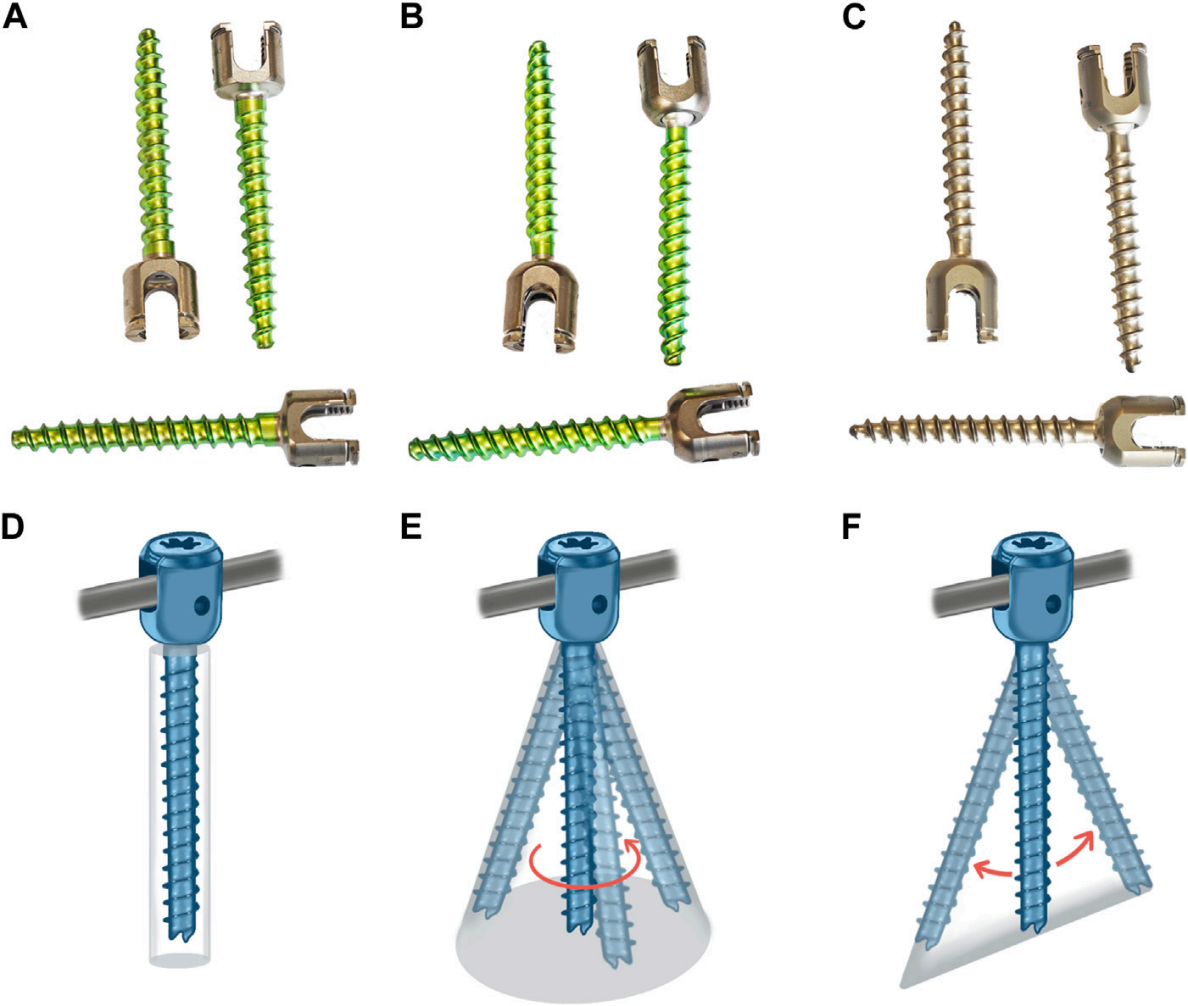
Three different pedicle screws and their schematic diagrams: **(A)** FAPS; **(B)** PAPS; **(C)** UPPS; **(D)** FAPS; **(E)** PAPS; **(F)** UPPS.

To ensure the overall biomechanical advantage of internal fixation and facilitate minimally invasive surgical operations, we designed a new uniplanar pedicle screw (UPPS) (Figure 1). The screw head of UPPS has a limited range of motion within one plane while remaining fixed in other planes. Theoretically, the free movement of the screw head on the axial plane of the body does not sacrifice the stiffness of the entire internal fixation structure on the sagittal plane, and at the same time facilitates the insertion of the connecting rod. UPPS offers the advantages of both FAPS and PAPS and is ideal for minimally invasive posterior spinal surgery (Peck et al., 2021).

In addition, our previous conducted research using the finite element method and found that a four pedicle screws and two modified new intermediate screws (NIS) had similar biomechanical advantages compared to the six-pedicle screw configuration (Li et al., 2020; Guo et al., 2021). This configuration allows bilateral intermediate screws to reach the center of the injured vertebra, elevate the depressed endplate, and maintain its reduction position more efficiently than the traditional parallel configuration of pedicle screws. The new configuration can enhance internal fixation strength and enable simultaneous vertebroplasty and bone grafting procedures by surgeons. However, further research is needed to determine whether UPPS is applicable to this new configuration.

In this paper, the range of motion of the vertebral body in the T12-L1 and L1-L2 segments was measured using different internal fixation devices through biomechanical research methods. The biomechanical advantages of the UPPS internal fixation system were verified by comparing the differences in the vertebral body movements in the six spatial directions, including flexion, extension, left bending, right bending, left rotation, and right rotation. The following report presents the results of our research.

## 2 Materials and methods

### 2.1 Design and manufacture of uniplanar pedicle screws and new intermediate screws

The 3D model of UPPS was created using Solidworks software (Dassault Systèmes, Concord, MA, United States). This screw has an inner diameter of 4.1 mm and an outer diameter of 6 mm, and its hollow design enables minimally invasive insertion, with a 2 mm diameter hollow lumen. Unlike FAPS, the UPPS has a head-shank connection that allows for a ±30° range of motion on the body axis. Spot welding is used in the manufacturing process to minimize the movement of the head-shank in other directions.

The new intermediate screw was developed based on the USS^®^ cannulated schanz screw. The NIS design features threads situated at the one-third shank, with a smooth section in the middle.

### 2.2 Specimen preparation

A total of 24 normal fresh cadaveric spine specimens (T12-L2) were carefully selected for this study. Each specimen was examined visually and with X-ray observation to ensure that no damage to the functional unit of the spine or abnormal bony structures were present. After removing the superficial muscle, fat and soft tissue, the inter-articular ligament and intervertebral disc structure were preserved. The specimens were then wrapped in double-layer plastic bags and stored at −20°C for later use. Prior to testing, the specimens were thawed at room temperature for 5 h. To create a model of fracture, cuneiform osteotomy was performed on the L1 vertebral body. The sample, test purpose, process, and post-test treatment process were approved by the Guangdong Provincial Medical Biomechanics Laboratory and related units, and all the procedures were carried out in accordance with the ethics guidelines established by the Chinese PLA General Hospital, Beijing, China.

### 2.3 Groups

**FIGURE 2:**
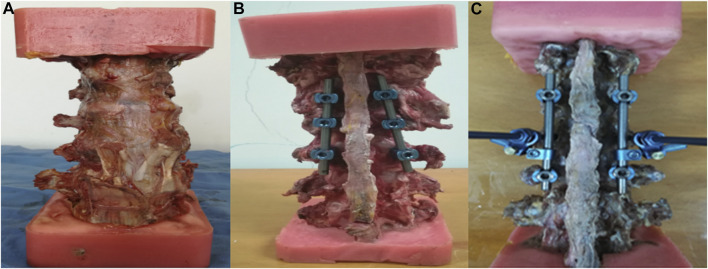
Show the spinal specimen and samples after internal fixation procedures. **(A)** Normal fresh cadaveric spine specimens; **(B)** 6-screw configuration; **(C)** 4-screw/2-NIS configuration.

The biomechanical test comprised two parts. In the first part, we aimed to investigate the biomechanical differences among UPSS, FAPS, and PAPS for the short-segment 6-screw configuration, respectively. The three groups were labeled as follows:1) 6-UPPS 2) 6-FAPS 3) 6-PAPS. The second part of the experiment was designed to compare the biomechanical differences of the three types of pedicle screws in the 4-screw configuration with two-NIS. This was labeled as follows: 1) 4-UPPS/2-NIS; 2) 4-FAPS/2-NIS; 3) 4-PAPS/2-NIS (Figure 2). To ensure proper experimental design and grouping, we equally divided the 24 specimens into 6 groups, with 4 samples in each group.

### 2.4 Surgical operation

In the first part of the experiment, 6 pedicle screws were inserted into each specimen, with two screws placed in each of the T12, L1, and L2 segments. The screws were all 6.0 mm in diameter and 45 mm in length and connected longitudinally by connecting rods without cross-links. A standard surgical procedure was followed by the same experienced surgeon, adhering strictly to the manufacturer’s specifications for screw placement.

In the second part of the experiment, 4 pedicle screws were inserted into each specimen, with 2 screws placed in each of the T12 and L2 levels. The newly developed NIS screw was inserted laterally at the L1 level, which is hollow and has inner and outer diameters of 5.2 and 6 mm, respectively. The NIS screw was connected to the longitudinal rod connecting the T12 and L1 pedicle screws through a connecting device. The rest of the surgical procedure was the same as in the first part, and was performed by a deputy chief physician with extensive surgical experience.

**FIGURE 3:**
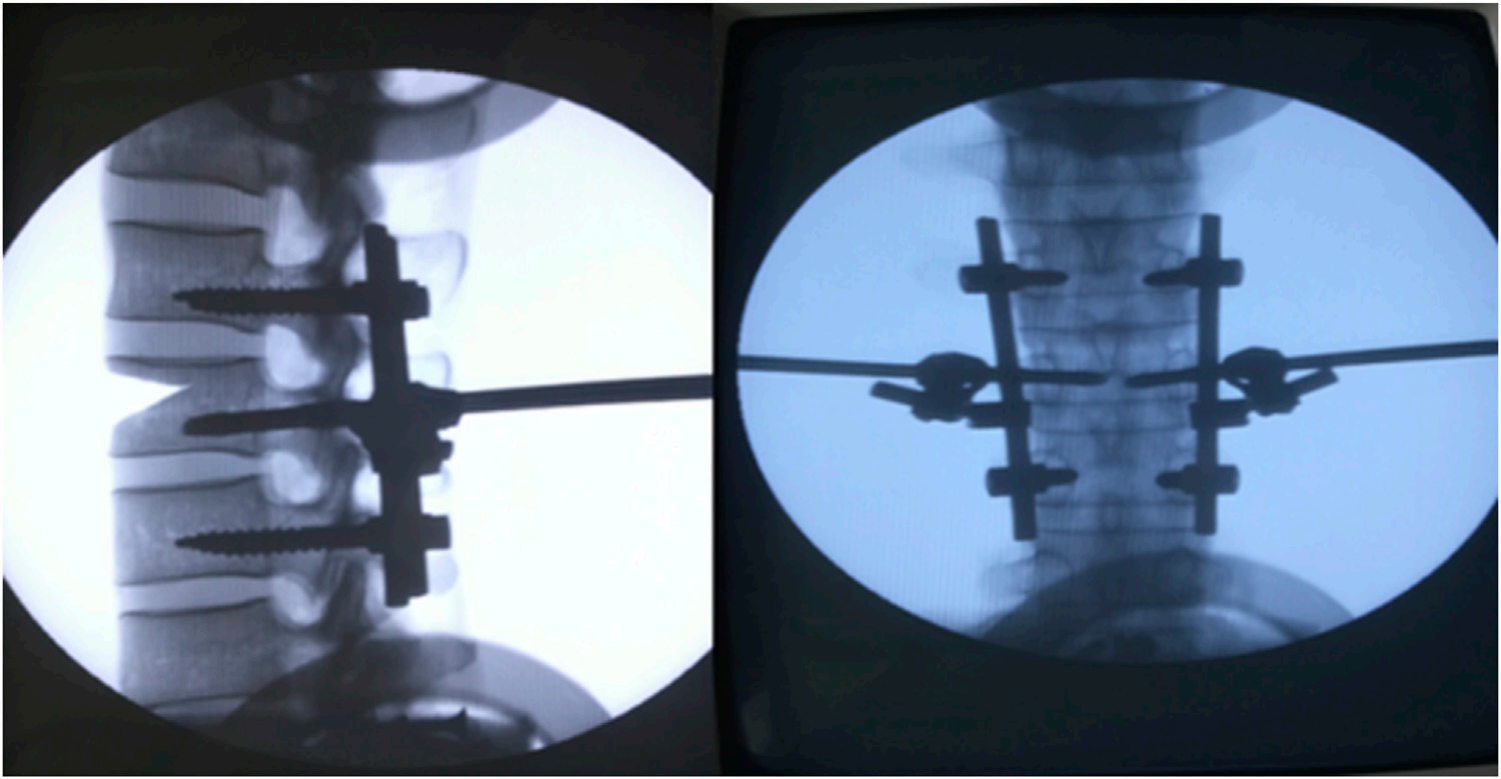
X-rays were used to confirm the success of the internal fixation insertion.

After the placement of internal fixation for each specimen was completed, the effectiveness of the placement was confirmed by taking an X-ray (Figure 3).

### 2.5 Biomechanical tests

To maintain the mechanical properties of fresh specimens and ensure accurate test results, the entire test procedure for each specimen must be completed within 8 h. In the event of ligament rupture, fracture, or intervertebral disc herniation, testing of the affected specimen must be discontinued, and a replacement specimen must be used for subsequent testing.

The testing process is to place the specimen on the pure force couple loading table, fix the L2 vertebral body, and allow the T12 vertebral body to move freely. The T12 vertebral body is connected with the force couple loading link, and the 8NM pure force couple is uniformly loaded on the six degrees of freedom of flexion, extension, left bending, right bending, left rotation, and right rotation. The motion and force characteristics of the specimen are evaluated under the same loading conditions.

**FIGURE 4:**
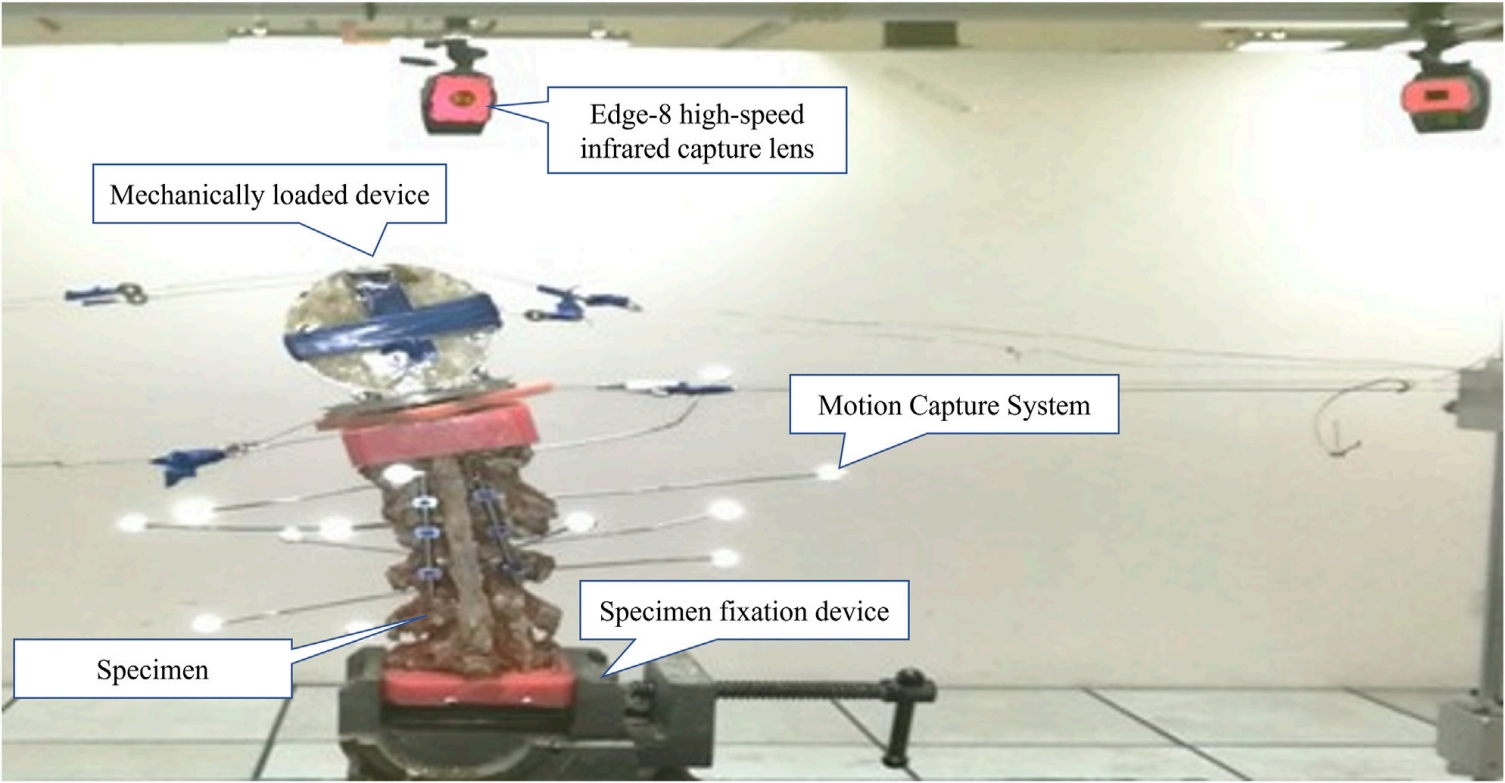
Sample in motion capture system.

The motion capture system and EVaRT software (Motion Analysis Company, United States) were used to measure the absolute value of range of motion (ROM) of each segment of the specimen (Figure 4). The system employs the Edge-8 high-speed infrared capture lens, which can achieve fast (response time <0.001s) and high-precision (0.001 mm) capture of Marker point space coordinates.

### 2.6 Statistical analysis

Statistical analysis was performed using SPSS 19.0 software, and the results were presented in the form of mean ± standard deviation. Statistical differences between groups were compared using one-way ANOVA. Significance was defined as *p* < 0.05.

## 3 Results

### 3.1 Spinal ROM in 6-screw configuration group

During the biomechanical testing of the 6-screw configuration, no instances of ligament rupture or bony structure compromise were observed in any of the specimens.

#### 3.1.1 ROM of T12-L1 level

**FIGURE 5:**
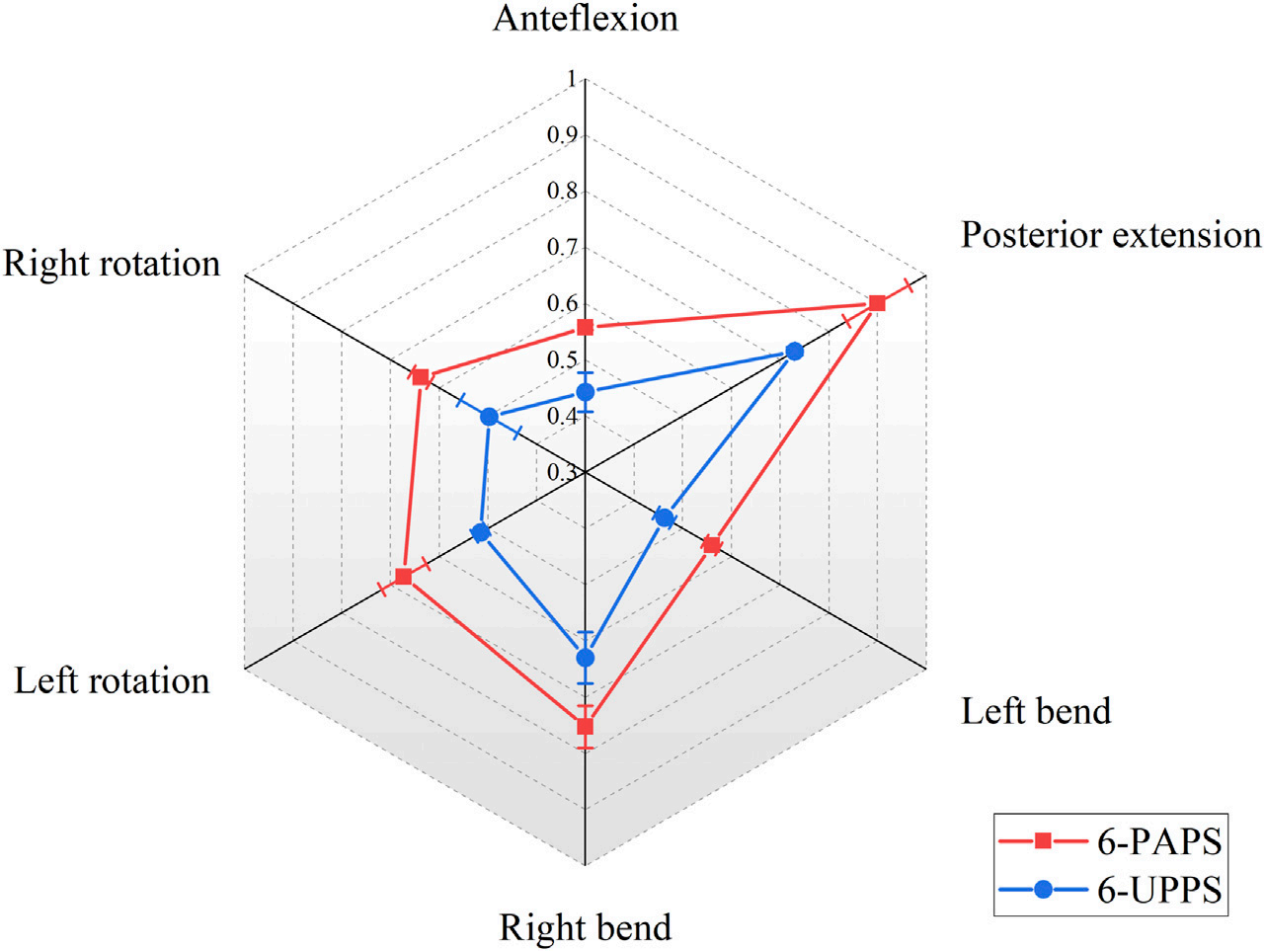
Show the ROM (°) of the T12-L1 segment in 6-screw configurations.

At the T12-L1 level, with the 6-screw configuration composed of UPPS, the ROM of the spine in six degrees of freedom of flexion, extension, left curvature, right curvature, left rotation, and right rotation was 0.44° ± 0.04°, 0.73° ± 0.01°, 0.46° ± 0.02°, 0.63° ± 0.05°, 0.52° ± 0.01°, and 0.50° ± 0.06°, respectively. These results are significantly better than the 6-screw configuration composed of PAPS at all degrees of freedom levels (*p* < 0.01) (Figure 5). Besides, in specimens using FAPS, the ROM at the T12-L1 level was also found to be superior to those using PAPS (Supplementary Table S1).

#### 3.1.2 ROM of L1-L2 level

**FIGURE 6:**
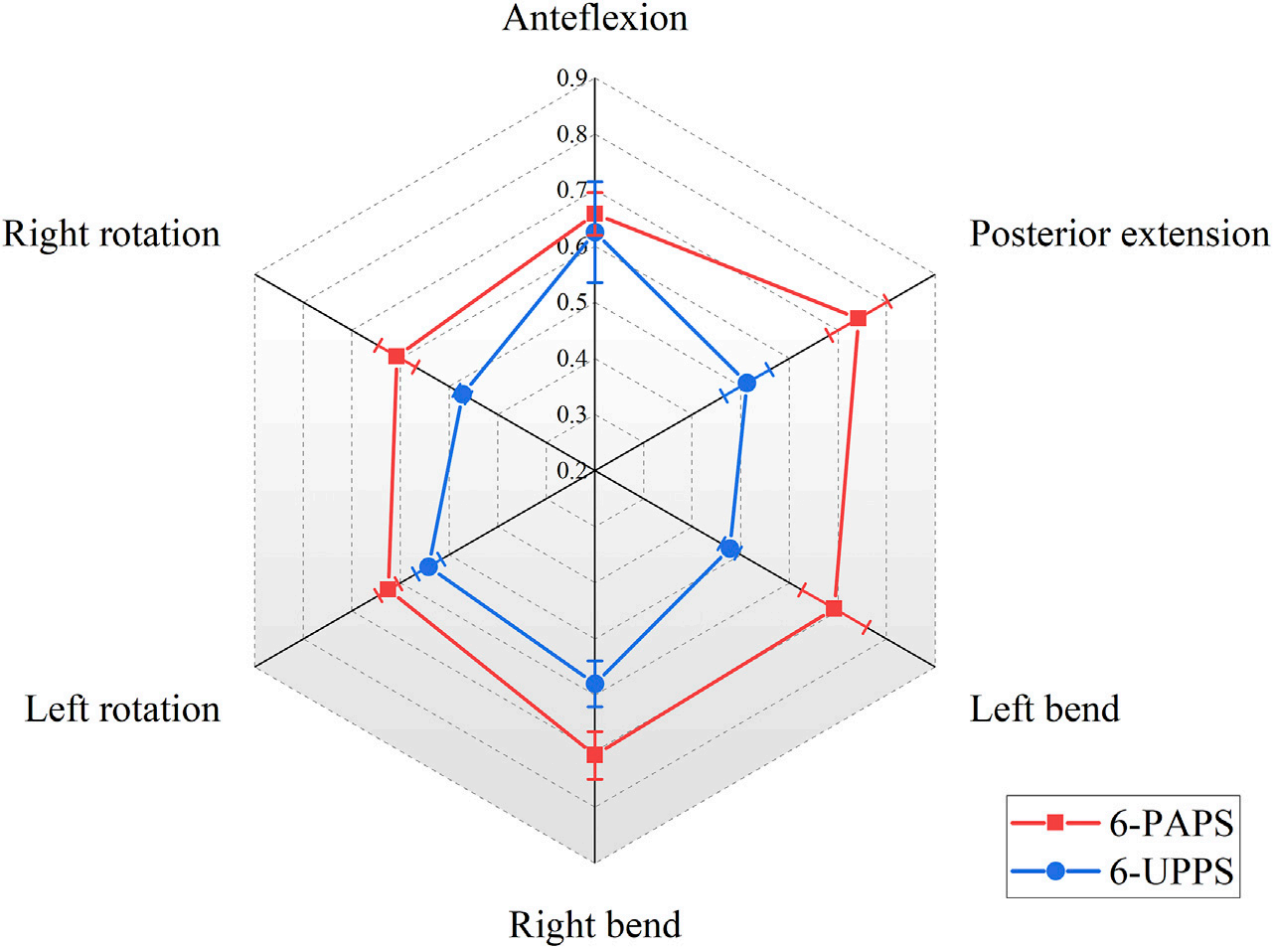
Show the ROM (°) of the L1-L2 segment in 6-screw configurations.

At the L1-L2 level, with the 6-screw configuration composed of UPPS, the ROM of the spine under loads of flexion, extension, left curve, right curve, left rotation, and right rotation were 0.63° ± 0.09°, 0.51° ± 0.05°, 0.48° ± 0.02°, 0.58° ± 0.04°, 0.54° ± 0.03°, and 0.47° ± 0.01°, respectively. Similar to the T12-L1 level, this result was also significantly better than the 6-screw configuration composed of PAPS at all degrees of freedom levels (*p* < 0.01) (Figure 6). The specimens utilizing FAPS demonstrated superior spinal range of motion at the L1-L2 level compared to those using PAPS as well (Supplementary Table S2).

### 3.2 Spinal ROM in 4-screw/2-NIS configuration group

During the biomechanical testing of the 4-screw/2-NIS configuration, no significant damage or destruction of load-bearing structures such as ligaments or bony structures were observed.

#### 3.2.1 ROM of T12-L1 level

**FIGURE 7:**
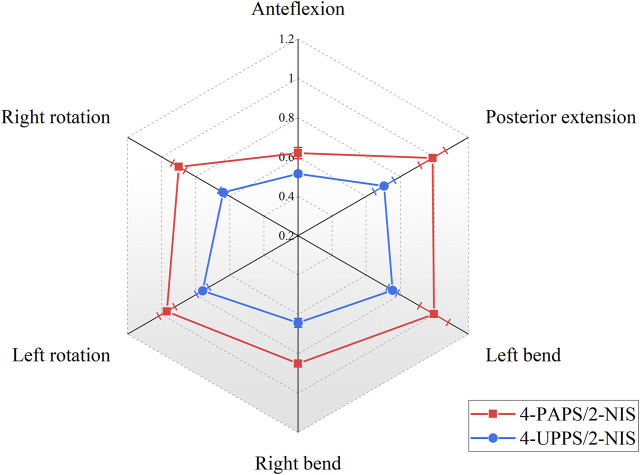
Show the ROM (°) of the T12-L1 segment in 4-screw/2-NIS configurations.

The internal fixation configuration composed of 4 UPPS and 2 NIS made the ROM of the spine model at the T12-L1 level under flexion, extension, left bending, right bending, left rotation, and right rotation force couples to be 0.52° ± 0.01°, 0.71° ± 0.06°, 0.76° ± 0.03°, 0.64° ± 0.02°, 0.76° ± 0.04°, and 0.64° ± 0.02°, respectively. The results were superior to the configuration consisting of 4 PAPS/2 NIS (*p* < 0.01) (Figure 7). In addition, ROM of the spine at the T12-L1 level in specimens that utilized 4-FAPS/2-NIS was found to be superior compared to those utilizing 4-PAPS/2-NIS (Supplementary Table S3).

#### 3.2.2 ROM of L1-L2 level

**FIGURE 8:**
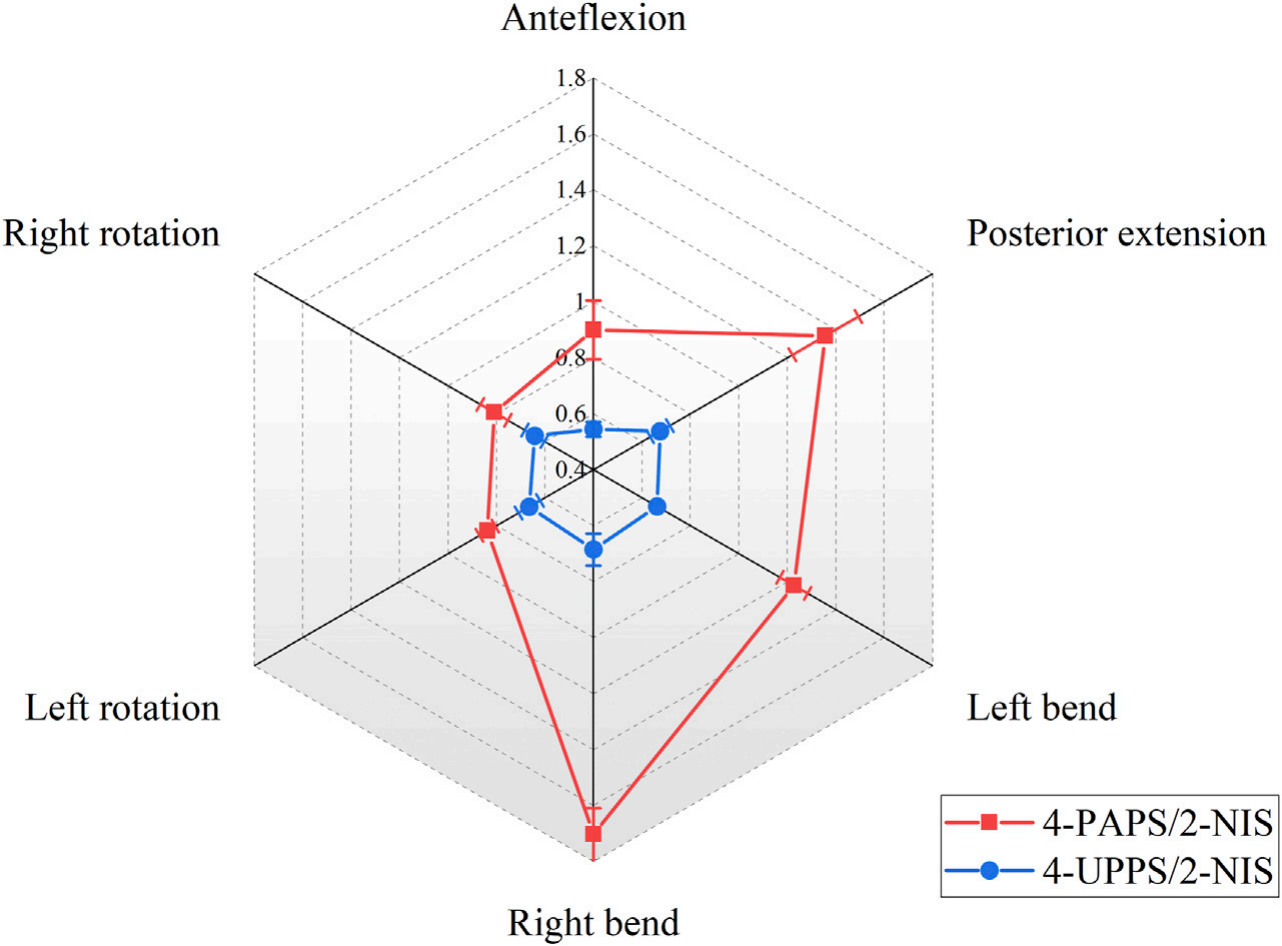
Show the ROM (°) of the L1-L2 segment in 4-screw/2-NIS configurations.

At the L1-L2 level, the 4-UPPS/2-NIS configuration also showed better spinal stability than 4-PAPS/2-NIS (*p* < 0.01) (Figure 8). The ROM under flexion, extension, left bending, right bending, left rotation and right rotation couples were 0.55° ± 0.03°, 0.68° ± 0.04°, 0.66° ± 0.01°, 0.69° ± 0.06°, 0.67° ± 0.04°, and 0.64° ± 0.04°, respectively. Additionally, according to the results of the experiment, ROM of the spine at the L1-L2 level in specimens that utilized 4-FAPS/2-NIS was also found to be superior compared to those utilizing 4-PAPS/2-NIS (Supplementary Table S4).

## 4 Discussion

This study provides evidence that UPPS confers unquestionable biomechanical advantages in spinal surgery. Biomechanical testing of both 6-screw and 4-screw configurations revealed no damage or destruction to load-bearing structures such as ligaments and bones. Additionally, we measured the ROM of each spine segment in six degrees of spatial freedom. ROM partially reflects the mechanical stability of the fused vertebral body in biomechanical experiments. Our findings indicate that the use of UPPS screws results in superior biomechanical stability compared to PAPS models. These results have significant implications for the treatment of spinal pathologies with minimally invasive surgery, expanding the range of available surgical options.

Minimally invasive spine surgery minimizes the incidence of postoperative low back pain and weakness in patients with open posterior spine surgery. Clinical application also verified the protection effect of the back muscles by the minimally invasive spine surgery. Kim et al. compared the trunk muscle strength of patients who underwent open posterior spinal fixation and those who underwent percutaneous internal fixation and found that the lumbar spine extension improved by more than 50% in patients who underwent percutaneous fixation, but not in patients who underwent open surgery (Kim et al., 2005). Kawaguchi et al. (1994) performed muscle biopsies on patients who underwent spinal revision surgery and observed atrophy of both type I and type II muscle fibers, extensive branching of fibrous tissue, and a “moth-eaten” appearance (Zhao et al., 2000). This behavior is caused by muscle compression, similar to the use of pneumatic tourniquets in extremity surgery. Additionally, denervation is thought to be the mechanism of muscle degeneration and atrophy after traditional open surgery. Multifidus, a muscle with a single-segmental distribution of nerves, is particularly vulnerable to injury (Hodges et al., 2006). Prolonged stretches can cause damage at the neuromuscular junction, leading to muscle denervation and postoperative muscle degeneration and atrophy.

Given the advantages of minimally invasive surgery, a matching pedicle screw design is required. The conventional FAPS is secured between the threaded section and the head, which provides high mechanical stability after vertebral body fusion. Our research further supported this claim. At levels of T12-L1 and L1-L2, FAPS screws had the least ROM values across all setups, indicating greater biomechanical stability. However, the use of FAPS requires precise alignment of the pedicle screw caps of each spinal segment on the same horizontal line to ensure insertion of the connecting rod. Unfortunately, the limited exposure of the minimally invasive surgical field and the challenging passage of the rod make it difficult to perform this task. Moreover, the already constrained operating environment is made even more constrained by the employment of the screw sleeve throughout the surgical procedure. This leads to prolongation of operation time and poor treatment outcomes in some patients. Thus, the benefits of minimally invasive surgery may be outweighed by the challenges posed by the FAPS system.

In 2001, Foley et al. (2001) have developed the PAPS system. Using the geometric trajectory principle, the connecting rod can be inserted into the deep muscle precisely and conveniently. It has undergone revolutionary changes and is currently the most widely used pedicle fixation device for minimally invasive posterior spine surgery. However, due of the mobility between the PAPS head and threaded component, the total configuration’s sagittal mechanical strength is decreased. Its surgical impact is also less than that of FAPS since it cannot be employed as a tool for fracture reduction and lacks intervertebral compression and distraction capabilities.

This study demonstrated that the ROM of the spine was the largest in the configuration composed of PAPS, indicating that the stability of the spine was worse. This is consistent with other research findings. Palmisani reported in a retrospective study that the use of polyaxial pedicle screws which are less rigid and might therefore increase the risk of loss of correction with time (Palmisani et al., 2009). A study by Shim also found that the use of PAPS is not conducive to restoring the height of the anterior column of the vertebral body and correcting kyphosis (Shim and Seo, 2022). A previous finite element study of ours also confirmed that the stabilization of the spine was worse with PAPS than with FAPS. In addition, during flexion and extension of the spine, the overall von Mises stress of the internal fixation using PAPS was higher, indicating that the biomechanical performance of the internal fixation was poor (Li et al., 2020).

UPPS was designed to address the fixation challenges encountered during minimally invasive spine surgery, particularly in situations where the use of PAPS is not desirable from a biomechanical standpoint. Our findings support the notion that UPPS offers superior biomechanical benefits compared to PAPS. Although our experimental data show that FAPS has higher biomechanical stability than UPPS, there might not be a significant difference between the two in clinical practice. Yebin confirmed that the therapeutic effects of UPPS and FAPS were comparable in the follow-up period of 12–18 months through a retrospective research of 204 patients. Patients treated with UPPS experienced less intraoperative bleeding and spent less time in the hospital. Additionally, when it comes to restoring the anterior vertebral body’s height following surgery, UPPS and FAPS have comparable results (Ye et al., 2022). This demonstrates that UPPS combines the advantages of FAPS and PAPS, ensuring sufficient biomechanical stability while facilitating the operation and reducing the risk of postoperative complications.

The biomechanics benefits of UPPS have also been supported by earlier investigations. Ye and Luo compared the ultimate load of FAPS, UPPS, and PAPS for internal fixation failure in static and dynamic biomechanical tests. They confirmed that UPPS has better axial mechanical stiffness than PAPS, which can reduce the risk of loss of reduction (Ye et al., 2017). The use of long segmental fixation for spinal fusion in this work, however, raises concerns about multi-segment damage and aberrant stress distribution. Liu et al. (2019) tested three types of screws and recorded two parameters that affect the retentive force including the tilt angle and the nut tightening torque. They found that the tulip-rod interface of FAPS frequently has a tilt angle, and this greatly reduces the retentive force. Therefore, Serhan et al. (2010) advocated for the use of polyaxial or uniplanar screws at the distal end of long spinal constructs since these screws increase the strength of the rod-tulip interface, and tilt angle occurs frequently at the distal end of long spinal constructs.

Intermediate screw have been developed to facilitate vertebroplasty and reduction maneuver (Baaj et al., 2011; Wang et al., 2012; Liao et al., 2017; Basaran et al., 2019). According to our prior article’s finite element analysis, the configuration of employing two new intermediate screws instead of two pedicle screws in the center can give appropriate biomechanical strength for the treatment of vertebral fractures (Li et al., 2020; Guo et al., 2021). In this paper, we continued our prior study by conducting biomechanical experiments using the newly designed intermediate screw. The results demonstrated that the 4UPPS/2NIS configuration was superior to 4PAPS/2NIS, consistent with the findings of the 6-screw configuration. In addition, the new intermediate screws offer benefits for both vertebroplasty and fracture reduction simultaneously. The new intermediate screw is placed in a manner, that is, more outside-in than typical pedicle screws, which enables it to reach the center of the fractured vertebral body and elevate the compressed endplate for better maintenance of reduction. Additionally, the newly created NIS nail features a lateral window at the distal end, that is, practical for bone grafting or filling with bone cement, enabling concurrent vertebroplasty during the procedure. By contrast, the traditional use of short-segment fixation requires the middle pedicle screw to be pulled out for bone cement filling or bone grafting after fracture reduction, then reinserted after vertebroplasty, which interrupts the normal operation and prolongs the operation time (Chen et al., 2014; Li et al., 2016). Our designed NIS simplifies the surgical procedure by allowing for vertebroplasty through the lateral window after reduction and fixation.

There are several limitations to this study that need to be addressed. Firstly, the biomechanical specimens used in this study were derived from normal spines, and the biomechanical characteristics of pathological spines, such as those with osteoporosis, may differ. Secondly, this study did not test the ultimate load of internal fixation, which could provide additional information on the stability of the constructs. Additionally, there are various similar screws available in clinical practice, but this study only compared one type of FAPS and PAPS. These are issues that warrant more study.

## 5 Conclusion

The biomechanical test findings revealed that the configuration employing the innovative UPPS had strong biomechanical benefits and assured spine stability. UPPS combines the biomechanical advantages of FAPS with the ease of use of PAPS. It is an optional minimally invasive internal fixation device for the treatment of thoracolumbar fractures.

## Data Availability

The original contributions presented in the study are included in the article/Supplementary Material, further inquiries can be directed to the corresponding authors.
