# Supplementary material for: Novel uniplanar pedicle screw systems applied to thoracolumbar fractures: a biomechanical study
Source: Front Bioeng Biotechnol. 2023 May 30;11:1172934. doi: 10.3389/fbioe.2023.1172934 (PMC10267819; doi:10.3389/fbioe.2023.1172934)
Supplement: Supplementary file 1 [file Table1.DOCX]

Supplementary Material

Novel Uniplanar Pedicle Screw Systems applied to thoracolumbar fractures: A Biomechanical study

Jiang Yuheng^123#^, Cui Xiang^12#^, Ji Wei^1#^, Li Jia^12^, Shi Yanli^4^, Zhao Jingxin^12^, Wang Junsong^12*^, Tang Peifu^12*^, Zhang Wei^12*^

*** Correspondence:** Wang Junsong: [waterandfood2022@163.com](mailto:waterandfood2022@163.com); Tang peifu: [pftang301@126.com](mailto:pftang301@126.com); Zhang Wei: [bszw@hotmail.com](mailto:bszw@hotmail.com)

# Supplementary Tables

| **T12-L1** | **anteflexion (**°**)** | **Posterior extension(**°**)** | **Left bend (**°**)** | **Right bend (**°**)** | **Left rotation (**°**)** | **Right rotation (**°**)** |
| --- | --- | --- | --- | --- | --- | --- |
| **6-PAPS** | 0.56±0.01 | 0.90±0.06 | 0.56±0.01 | 0.75±0.04 | 0.67±0.05 | 0.64±0.02 |
| **6-UPPS** | 0.44±0.04 | 0.73±0.01 | 0.46±0.02 | 0.63±0.05 | 0.52±0.01 | 0.50±0.06 |
| **6-FAPS** | 0.38±0.04 | 0.61±0.01 | 0.39±0.02 | 0.53±0.06 | 0.44±0.03 | 0.36±0.03 |

**Supplementary Table 1.** ROM of T12-L1 level in 6-screw configurations

| **L1-L2** | **anteflexion (**°**)** | **Posterior extension(**°**)** | **Left bend (**°**)** | **Right bend (**°**)** | **Left rotation (**°**)** | **Right rotation (**°**)** |
| --- | --- | --- | --- | --- | --- | --- |
| **6-PAPS** | 0.66±0.04 | 0.74±0.06 | 0.69±0.07 | 0.71±0.04 | 0.63±0.02 | 0.61±0.04 |
| **6-UPPS** | 0.63±0.09 | 0.51±0.05 | 0.48±0.02 | 0.58±0.04 | 0.54±0.03 | 0.47±0.01 |
| **6-FAPS** | 0.36±0.09 | 0.42±0.02 | 0.33±0.01 | 0.52±0.01 | 0.46±0.04 | 0.39±0.08 |

**Supplementary Table 2.** ROM of L1-L2 level in 6-screw configurations

| **T12-L1** | **anteflexion (**°**)** | **Posterior extension(**°**)** | **Left bend (**°**)** | **Right bend (**°**)** | **Left rotation (**°**)** | **Right rotation (**°**)** |
| --- | --- | --- | --- | --- | --- | --- |
| **4-PAPS/ 2NIS** | 0.62±0.03 | 0.99±0.08 | 1.00±0.08 | 0.85±0.02 | 0.97±0.05 | 0.90±0.04 |
| **4-UPPS/ 2-NIS** | 0.52±0.01 | 0.71±0.06 | 0.76±0.03 | 0.64±0.02 | 0.76±0.04 | 0.64±0.02 |
| **4-FAPS/ 2-NIS** | 0.33±0.03 | 0.54±0.02 | 0.64±0.02 | 0.55±0.01 | 0.64±0.02 | 0.47±0.02 |

**Supplementary Table 3.** ROM of T12-L1 level in 4-screw/2-NIS configurations

| **L1-L2** | **anteflexion (**°**)** | **Posterior extension(**°**)** | **Left bend (**°**)** | **Right bend (**°**)** | **Left rotation (**°**)** | **Right rotation (**°**)** |
| --- | --- | --- | --- | --- | --- | --- |
| **4-PAPS/ 2NIS** | 0.90±0.10 | 1.36±0.14 | 1.23±0.06 | 1.70±0.09 | 0.84±0.03 | 0.81±0.06 |
| **4-UPPS/ 2-NIS** | 0.55±0.03 | 0.68±0.04 | 0.66±0.01 | 0.69±0.06 | 0.67±0.04 | 0.64±0.04 |
| **4-FAPS/ 2-NIS** | 0.46±0.03 | 0.54±0.04 | 0.50±0.04 | 0.49±0.02 | 0.58±0.03 | 0.54±0.03 |

**Supplementary Table 4.** ROM of L1-L2 level in 4-screw/2-NIS configurations
